# Supplementary figures and images for: Investigation of Blood Coagulation Using Impedance Spectroscopy: Toward Innovative Biomarkers to Assess Fibrinogenesis and Clot Retraction
Source: Biomedicines. 2022 Jul 29;10(8):1833. doi: 10.3390/biomedicines10081833 (PMC9404805; doi:10.3390/biomedicines10081833)

## Slide 1
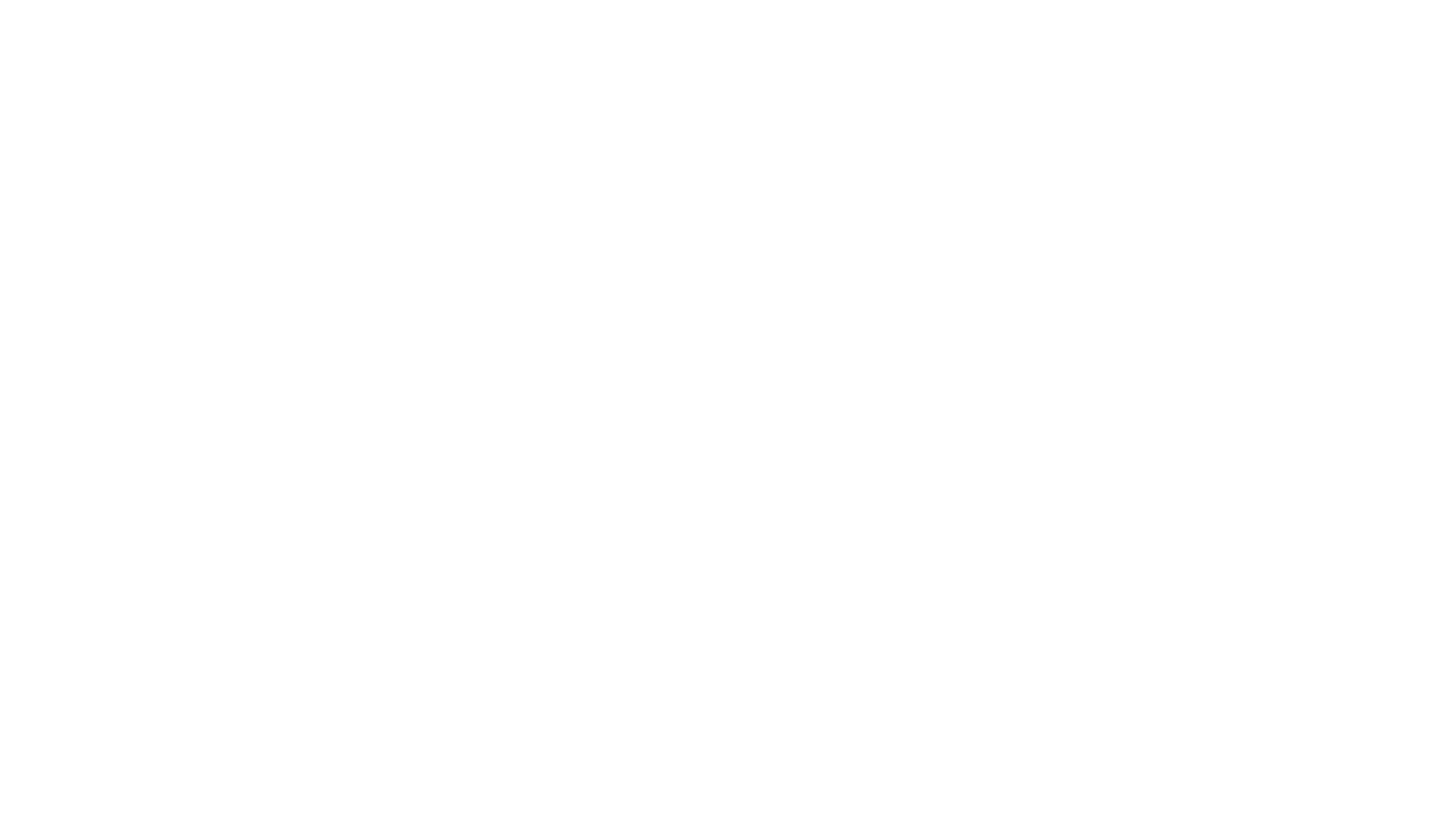

Supplement: Supplementary file 1 [file biomedicines-10-01833-s001.zip › biomedicines-1811588-supplementary.ppsm]
